# MitSorter: a standalone tool for accurate discrimination of mtDNA and NuMT ONT reads based on differential methylation

**DOI:** 10.1093/bioadv/vbaf135

**Published:** 2025-07-10

**Authors:** Sharon Natasha Cox, Angelo Sante Varvara, Graziano Pesole

**Affiliations:** Department of Biosciences, Biotechnology and Environment, University of Bari Aldo Moro, Bari 70125, Italy; Department of Biosciences, Biotechnology and Environment, University of Bari Aldo Moro, Bari 70125, Italy; Department of Biosciences, Biotechnology and Environment, University of Bari Aldo Moro, Bari 70125, Italy; Institute of Biomembranes, Bioenergetics and Molecular Biotechnologies, National Research Council (CNR), Bari 70126, Italy; Consorzio Interuniversitario Biotecnologie, 34148 Trieste, Italy

## Abstract

**Motivation:**

The accurate differentiation between mitochondrial DNA (mtDNA) and nuclear mitochondrial DNA segments (NuMTs) is a critical challenge in studies involving mitochondrial disorders. Mapping the mtDNA mutation spectrum and quantifying heteroplasmy are complex tasks when using next-generation sequencing methods, mostly due to NuMTs contamination in data analysis.

**Results:**

Here, we present a novel, easy-to-use standalone command-line tool designed to reliably discriminate long reads originated by either mtDNA or NuMTs and generated by Oxford Nanopore Technologies (ONT) sequencing based on the known lack of CpG methylation in human mtDNA. MitSorter aligns the reads to the mitochondrial genome incorporating base modification calls directly from raw POD5 files. The resulting BAM file is then partitioned into two separate BAM files: one containing unmethylated reads and the other containing methylated reads. We show that MitSorter analysis can provide a more accurate landscape of the mtDNA mutation profile. We describe here the tool's features, computational framework, validation approach, and its potential applications in other genomic research areas.

**Availability and implementation:**

Source code and documentation, are available at https://github.com/asvarvara/MitSorter.

## 1 Introduction

### 1.1 ONT sequencing and relevance to epigenetics investigation

Next-generation sequencing (NGS) technologies have revolutionized genomic analysis by offering unprecedented levels of coverage and throughput (Satam *et al.* 2023). Oxford Nanopore Technologies (ONT) have taken this revolution even further, enabling the reconstruction of the primary sequence with the identification of single nucleotide polymorphisms (SNPs), indels, and structural variations (SVs), thus providing a genome-wide resolution of base modifications in a single sequencing run of native DNA, thereby bringing the analysis to a higher level of completeness. This emerging sequencing platform enables a direct, real-time reading of long DNA or RNA molecules by decoding fluctuations in electrical current as nucleic acids pass through a protein nanopore (Madoui *et al.* 2015). The resulting signal is interpreted to provide the specific DNA or RNA sequence, along with base modifications in native molecules (Jain *et al.* 2016).

This methodology represents a revolutionary advancement in epigenetics, particularly in the study of stable epigenetic modification like 5-methylcytosine (5mC) and 5-hydroxymethylcytosine (5hmC). The analysis of native ONT reads overcomes the biases introduced by sodium bisulfite treatment, PCR amplification, and whole-genome bisulfite sequencing (WGBS) in detecting 5mC, although the latest protocols have attempted to limit these pitfalls (Dai *et al.* 2024). The main limitations in WGBS include DNA damage due to the lengthy reaction times at high temperature and overestimation of the 5mC level due to biased fragmentation at C sites making these methods unsuitable for rapid detection or diagnostic applications. When comparing ONT with WGBS, the former offers several advantages, especially in GC-rich regions, due to its unbiased coverage and direct methylation detection (Guanzon *et al.* 2024).

### 1.2 Lack of CpG methylation in mitochondrial DNA

Mitochondrial DNA (mtDNA) is a small, double-stranded, circular genome of 16 569 bp in humans (Anderson *et al.* 1981). It is characterized by a unique organization and composition, as it is composed of a guanine-rich “Heavy” (H) strand and a cytosine-rich “Light” (L) strand and this confers strands asymmetry. The circular topology and asymmetric nucleotide composition of the two strands of mtDNA present specific challenges for sequencing and methylation analysis. Notably, the cytosine-rich L strand is subject to disproportionate fragmentation, resulting in higher sequencing coverage (Olova *et al.* 2018). Others point out that the circular structure of mtDNA hinders bisulfite conversion efficiency which leads to an overestimation of mtDNA methylation values (Liu *et al.* 2016). ONT long reads have demonstrated their effectiveness in mapping sporadic mtDNA deletions without the need for mtDNA amplification and fragmentation (Vandiver *et al.* 2022), and the phasing of these mutations provides an unbiased picture of SNPs and Indels (Vandiver *et al.* 2023).

Despite all these reports, the presence of mtDNA methylation has until recently remained controversial and a subject of debate. Some authors, using bisulfite sequencing and/or the 5mC and 5hmC DNA immunoprecipitation affinity-based methods which cannot provide a single base resolution, have claimed that modified cytosines are present preferentially in the D-loop region of mtDNA in samples extracted from the blood and cultured cells of both humans and mice, with methylation also occurring in non-CpG dinucleotides, as seen in bacterial genomes (Bellizzi *et al.* 2013). They suggest that these modifications could have a role in influencing the expression of transcripts of mtDNA. Concordant with these results, others suggested that DNA methyltransferase 1 (DNMT1) can translocate to the mitochondria, through a mitochondrial targeting sequence, and confirmed its presence in the mitochondrial matrix bound to mtDNA (Shock *et al.* 2011).

However, these results have been contradicted by several reports that conclusively demonstrate the lack of CpG methylation in mtDNA (Hong *et al.* 2013, Mechta *et al.* 2017, Matsuda *et al.* 2018). Notably, two independent research groups reached the same conclusion using distinct methodologies: an optimized bisulfite sequencing protocol and ONT long-read sequencing. Both groups consistently reported that mtDNA methylation levels are comparable to background noise (Bicci *et al.* 2021) or may range from 0.19% to 0.67% (Shao *et al.* 2023). Furthermore, both the groups asserted that the residual methylation signal likely originates from the interference of methylated nuclear mitochondrial DNA segments (NuMTs) misaligned to the mtDNA reference sequence. NuMTs are mtDNA-like sequences transferred to the nuclear genome over evolutionary time (Simone *et al.* 2011). In the fully assembled T2T-CHR13 human reference genome, 958 NuMTs have been identified, with lengths ranging from 28 to 14 855 bp (Tao *et al.* 2023) and spanning up to 90% of the entire mitochondrial genome (Ramos *et al.* 2011, Tao *et al.* 2023).

### 1.3 NuMTs and their impact

Along evolution, mtDNA-derived fragments integrated into the nuclear genome, and their high sequence similarity to mtDNA is a substantial challenge for NGS workflows, where DNA reads are aligned to reference genomes based on sequence identity. This high nucleotide similarity complicates the accurate identification of reads origin, leading to cross-mapping issues. Such cross-mapping can result in artefactual mtDNA variants when NuMT-derived reads are incorrectly aligned to the mitochondrial reference or false negatives when mtDNA reads are misaligned to nuclear NuMT loci (Duan *et al.* 2018). To address this issue, a commonly employed strategy for mitochondrial variant analysis involves isolating, amplifying, and sequencing the mitochondrial genome separately (Albayrak *et al.* 2016, Santibanez-Koref *et al.* 2019).

While this method is considered the gold standard for mtDNA genotyping, it is quite laborious, and many factors can still influence variant calling outcomes (Weerts *et al.* 2018). Additionally, the growing use of whole-genome sequencing (WGS) for large-scale diagnostics, where nDNA and mtDNA are analysed together, emphasizes the importance for accurate discrimination of mtDNA and NuMT reads. This is the first step to develop robust tools for accurately detecting mitochondrial mutations in high-throughput WGS pipelines (Turro *et al.* 2020).

Some bioinformatics tools for mitochondrial genome reconstruction rely on a double alignment, first on the mitochondrial reference sequence and then on the human genome build to discard NuMTs (Picardi and Pesole 2012). This approach may have some limitations because of the homologous overlapping sequences, making the cleaning process inaccurate. Our tool exploits the underlying biological differences between true mtDNA reads from NuMTs based on the different CpG methylation status which results in minimizing NuMTs contamination. ONT offers a unique advantage by enabling single-molecule direct detection of DNA methylation during sequencing. Based on the known lack of CpG methylation in human mtDNA, we developed a command-line tool to use the methylation status at read level as a reliable discriminator between mtDNA and NuMTs.

## 2 Methods

### 2.1 MitSorter pipeline

MitSorter has been applied to the updated and improved release of Genome In A Bottle (GIAB) human ONT data (HG002, HG003, HG004), which is freely available from EPI2ME (https://labs.epi2me.io/giab-2025.01/). This dataset represents a well-characterized family trio of Ashkenazi Jewish origin, widely used as a benchmark for variant calling and other genomics tools, owing to its comprehensive and extensively curated variant truth sets derived from both short- and long-read sequencing data. This widely used benchmark dataset enables accurate pipeline testing, as well as robust performance evaluation across different sequencing technologies. This dataset contains high-quality whole-genome ONT sequencing data generated using the latest R10.4.1 pore, which, in combination with Kit 14 chemistry, provides enhanced sequencing accuracy and performance.

**Figure 1.**
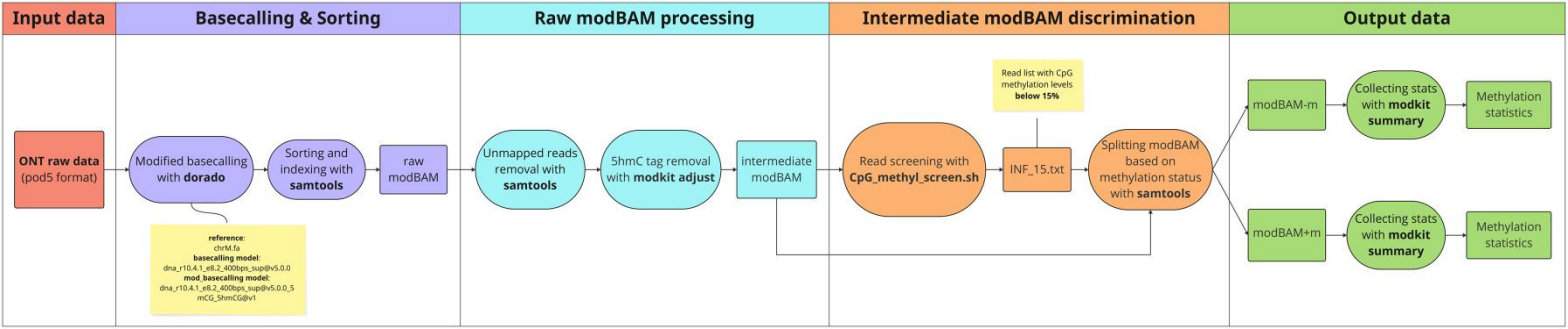
A schematic overview of MitSorter pipeline for processing and classifying modBAM files derived from ONT raw sequencing data. The pipeline starts with basecalling using Dorado from raw.pod5 data following with sorting and indexing using samtools, thus generating the modBAM raw file. This file undergoes filtering to remove unmapped reads and remove 5hmC-related tags via modkit adjust, resulting in the intermediate modBAM. Reads are then screened for CpG methylation levels using the custom script CpG_methyl_screen.sh, producing a list of low-methylation reads (INF_15.txt). Based on this list, the intermediate modBAM is split into two distinct files: modBAM-m (reads with <15% CpG methylation) and modBAM+m (reads with ≥15% CpG methylation). The final output includes methylation summary statistics for both partitions, generated with modkit summary.

A schematic overview of MitSorter pipeline is shown in Fig. 1. The pipeline starts with processing raw ONT POD5 files by the high-performance basecaller Dorado (v0.7.2). Basecalling has been executed in high-accuracy mode using the model dna_r10.4.1_e8.2_400bps_sup@v5.0.0, which supports standard base detection, alongside the methylation-aware model dna_r10.4.1_e8.2_400bps_sup@v5.0.0_5mCG_5hmCG@v1, optimized for detecting both 5mC and 5hmC modifications. Reads have been aligned to the recently published complete human mitochondrial reference genome under GenBank accession number CP068254.1 (Nurk *et al.* 2022). The output of Dorado is a raw modBam file, which incorporates modified-base information stored in the MM (modified-base probabilities) and ML (probability scores) tags, in accordance with section 1.7 of the SAM tags specification (https://samtools.github.io/hts-specs/SAMtags.pdf). These tags are generated by latest ONT basecallers to annotate modified-base data.

This raw modBam is processed further and the sorting and removal of unmapped reads are performed using samtools (v1.19.2). For downstream analysis, Modkit (v0.4.1) is employed with the adjust-mods subcommand to filter out 5hmC calls from the raw modBam file, only retaining 5mC modifications producing a modBAM file we named “intermediate”. A custom Bash script (CpG_methyl_screen.sh) is then used to process the intermediate modBam file and quantify CpG methylation levels for each read based on the information encoded in the MM and ML tags. MitSorter therefore splits the intermediate modBam file into two BAM files: one containing reads with CpG methylation levels below 15% (named modBAM−m) and another one containing reads with CpG methylation levels equal to or exceeding 15% (named modBAM+m).

The arbitrary choice of the 15% threshold was guided by the bimodal distribution of CpG read methylation percentages observed when analysing the intermediate modBAM files (Supplementary Fig. S1A, available as supplementary data at *Bioinformatics Advances* online) where the vast majority of reads (73 946; approximately 74%) show a methylation level below 15%. This process enables precise stratification of reads based on methylation levels, for use in downstream analyses. The final modBam +m and −m files undergo basic statistical analysis, including read counts, alignment metrics, and an overview of the modification tags present and the information is captured in the log for comprehensive reporting.

The MitSorter workflow has been generated through Snakemake (v8.27.1) management system to easily reproduce and parallelize the above-mentioned analysis steps. All necessary tools are provided by an *ad hoc* built Conda environment with required dependencies specified in our .YAML file.

### 2.2 Validation strategy

#### 2.2.1 Skew analysis

GC- and AT-skew were computed at the single-read level for the intermediate, the modBAM+m, and the modBAM−m files. These skew values were then averaged across all reads using a custom Bash script providing insight into strand composition biases potentially related to the read origin.

#### 2.2.2 Assessment of NuMT discrimination

To validate MitSorter’s ability to accurately distinguish true mtDNA reads from NuMTs, all reads in FASTQ format were extracted from the original intermediate modBAM file using samtools and aligned to the T2T reference genome with minimap2 (v.2.28-r1209), with newly identified NuMTs (Tao *et al.* 2023) and chrM masked.

#### 2.2.3 Variant calling comparison

Variant calling was performed using the newly developed web-based tool Mitopore optimized for long reads (Dobner *et al.* 2024). FASTQ files were generated from both the intermediate modBAM and the modBAM−m files using samtools fastq. The tool realigns reads to the mtDNA, generating a new BAM file aligned to the rCRS (NC_012920.1). This BAM file was then used as input for a second variant caller, mtDNA-Server 2 (Weissensteiner *et al.* 2024). Variant statistics were generated using bcftools (v1.12).

### 2.3 Identification and visualization of recent mtDNA insertions into the nuclear genome

To identify recent mtDNA insertions into nDNA, sequencing reads were extracted from modBAM+m files using samtools fastq. The resulting FASTQ files were aligned to the T2T reference genome with minimap (v.2.28-r1209) and filtered with samtools view using the -L option to retain only reads mapping to known NuMTs as defined in Tao *et al.* (2023). This process yielded two BAM files: one containing reads aligning to known NuMT loci, and another unknown NuMT loci. The resulting alignments were visualized using Circos (v0.69-8).

## 3 Results

We evaluated MitSorter using the latest release of the GIAB Ashkenazi trio dataset (https://labs.epi2me.io/giab-2025.01/). The analysis of the intermediate modBAM containing reads aligning to ChrM exhibited a consistent low CpG methylation level with a bimodal distribution across all subjects (Supplementary Fig. S1A, available as supplementary data at *Bioinformatics Advances* online). Considering all reads within the intermediate modBAM across the three tested samples (*N* = 99 897), the majority (69%, *N* = 68 947) fell into the lowest methylation bin (0.0%–5%), indicating that CpG sites in mtDNA are largely unmethylated. As methylation levels increased, the number of aligned reads decreased sharply, with only a small proportion placed in the intermediate methylation bins. A secondary peak at higher methylation levels (>55%) was observed, likely corresponding to NuMTs (Supplementary Fig. S1A, available as supplementary data at *Bioinformatics Advances* online).

MitSorter processes the intermediate modBAM file by stratifying reads into two distinct outputs: a modBAM−m (Supplementary Fig. S1B, available as supplementary data at *Bioinformatics Advances* online) and a modBAM+m (Supplementary Fig. S1C, available as supplementary data at *Bioinformatics Advances* online). Collectively, Modkit summary reports that the unsorted intermediate modBAM exhibited a mean 5mC fraction of 0.37 ± 0.13. The modBAM−m shows a 5mC fraction of 0.0022 ± 0.0003, while the modBAM+m of 0.63 ± 0.28 (Table 1). This analysis demonstrates that MitSorter can effectively distinguish between true unmethylated mtDNA reads and highly methylated NuMTs. This clear separation is further supported by the fact that reads in the modBAM−m file exhibited a uniform GC% of 44.37 as well as GC- and AT-skews, on average −0.357 and 0.100, respectively, corresponding to the known value for ChrM (Anderson *et al.* 1981). On the contrary, the modBAM+m showed values of GC%, GC-, and AT-skew of 40.50, −0.061, and 0.017, quite divergent from those observed in human mtDNA (Table 1).

**Table 1.** Basic alignment statistics of reads mapping to ChrM, along with a summary of methylated cytosine (5mC) levels in both intermediate and final processed BAM files (modBAM+m and modBAM−m).

	HG002	HG003	HG004	Mean	SEM	HG008N-P
Flow cells	PAW71238/PAW70337	PAY87794/PAY87954	PAY87778/PAY88428			PAU35760/PAQ93590/PAU23348
n° POD5 pass	149/156	116/108	102/114			668/764/771
Total reads (N°)	15 607 880	15 369 926	15 128 647	15 368 818	239 618	
**Intermediate modBAM**						
UNIQUE READS	14 392	39 270	28 710	27 457	12 486	33 179
Coverage ChrM	2604	6834	9427	6288	3444	11 694
GC%	42.82	43.92	43.77	43.50	0.60	44.20
GC skew	−0.190	−0.303	−0.288	−0.261	0.061	−0.312
AT skew	0.055	0.085	0.080	0.073	0.016	0.089
Read length	20 561	8093	9427	12 694	6846	9377
Unmodified C	1 423 002	3 065 330	2 275 117	2 254 483	821 358	4 610 155
5mC	1 524 983	1 161 405	1 043 940	1 243 443	250 795	858 579
Fraction 5mC (5mC/C + 5mC)	0.52	0.27	0.31	0.37	0.13	0.157
**modBAM+m**						
UNIQUE READS	8666	7403	6922	7664	901	5104
Coverage ChrM	1002	857	790	883	108	986
GC%	40.34	40.50	40.65	40.50	0.16	42.31
GC skew	−0.049	−0.071	−0.065	−0.061	0.011	−0.101
AT skew	0.015	0.019	0.019	0.017	0.003	0.029
Read length	31 016	29 414	29 060	29 830	1042	25 683
Unmodified C	783 487	728 541	648 423	720 150	67 922	414 897
5mC	1 531 861	1 149 917	1 035 258	1 239 012	260 014	868 403
Fraction 5mC (5mC/C + 5mC)	0.66	0.61	0.61	0.63	0.03	0.68
**modBAM-m**						
UNIQUE READS	5726	31 867	21 788	19 794	13 184	28 075
Coverage ChrM	1602	5977	4146	3908	2197	10 707
GC%	44.34	44.40	44.36	44.37	0.03	44.37
GC skew	−0.370	−0.351	−0.350	−0.357	0.011	−0.344
AT skew	0.105	0.099	0.098	0.100	0.004	0.099
Read length	4746	3148	3204	3699	907	6413
Unmodified C	632 850	2 335 582	1 637 531	1 535 321	855 955	4 169 731
5mC	1632	4791	3534	3319	1590	19 232
Fraction 5mC (5mC/C + 5mC)	0.0026	0.002	0.0022	0.0023	0.0003	0.0046
**Alignment to masked T2T**						
UNIQUE READS	8520	6956	6567	7348	1034	4747
Read Length	31 552	31 307	30 652	31 170	465	27 894
Overlap in modBAM+m N° (%)	8457 (99%)	6729 (97%)	6377 (97%)	7188 (98%)	1113	4368 (92%)
**mtDNA Variant Calls**						
N° variants Intermediate modBAM^a^	8831	8912	3416	7053	3150	6332
N° variants modBAM−m^a^	14	41	17	24	15	12
N° variants Intermediate modBAM^b^	9748	9738	9134	9540	352	9608
N° variants modBAM−m^b^	13	44	16	25	17	39

a Obtained using Mitopore.

b Obtained using mitoverse2.

Similar results were found when we tested MitSorter on normal pancreatic tissue (GIAB HG008 N-P, https://www.ncbi.nlm.nih.gov/sra/SRX26067867; Table 1) and in other 12 private samples tested (data not shown). The observed bimodal distribution, with most reads exhibiting low methylation and a secondary peak at high methylation levels, supports the tool’s ability to accurately classify and identify bona-fide mitochondrial reads based on their CpG methylation status.

To validate MitSorter’s ability to accurately distinguish true mtDNA reads from NuMTs, we extracted all reads in FASTQ format from the intermediate modBAM file and realigned them to the T2T reference genome, with all newly identified NuMTs (Tao *et al.* 2023) and chrM being masked. The realigned reads demonstrated a consistent overlap with those present in the modBAM+m file (mean 98% of reads), while exhibiting a marginal overlap in the modBAM−m (Table 1).

**Figure 2.**
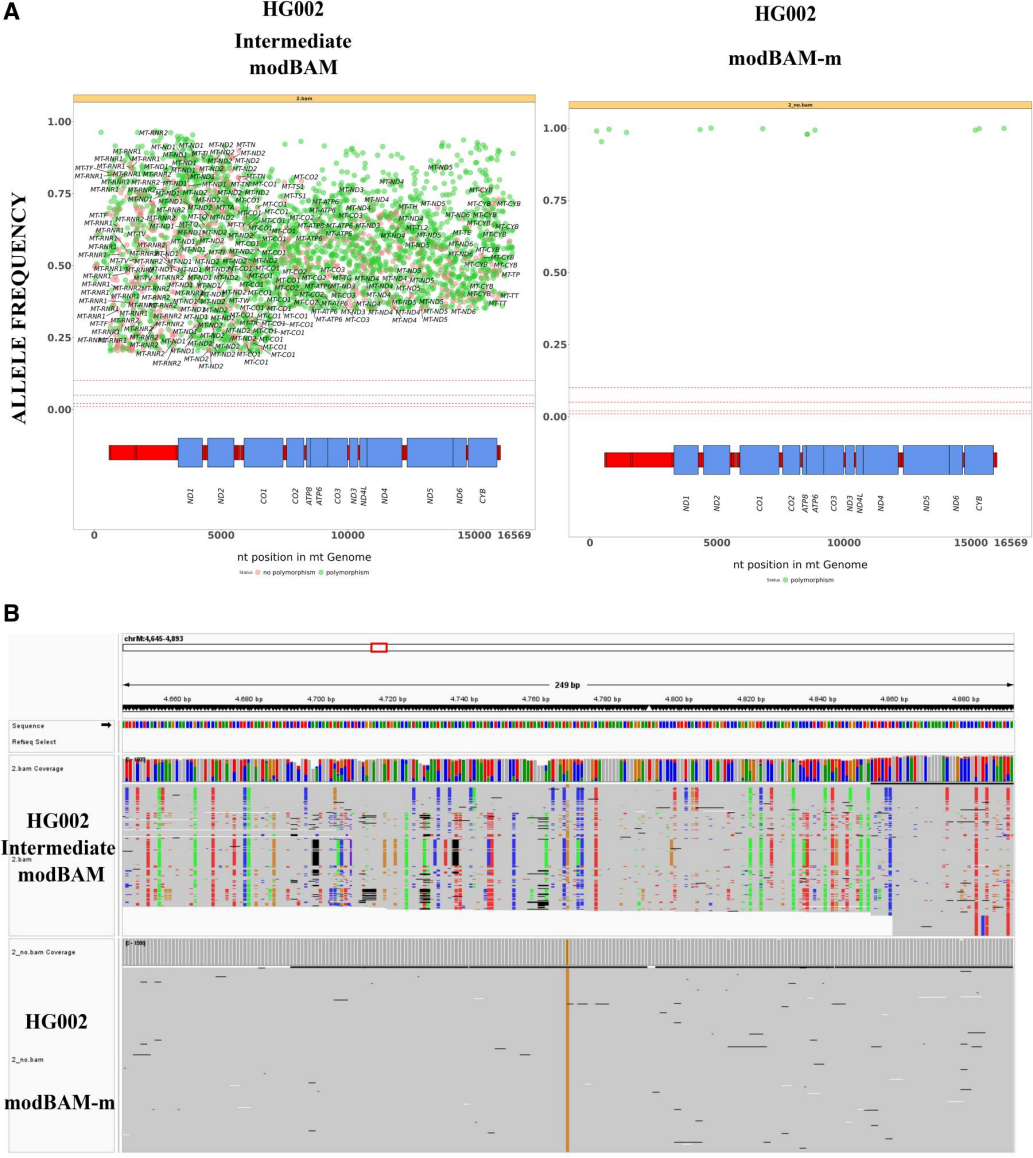
Impact of MitSorter methylation—aware read processing on mtDNA variant detection. (A) Allele frequency plots generated by Mitopore for HG002. The left plot displays variants identified from intermediate modBAM files, while the right plot shows variants generated from modBAM without methylated reads (modBAM-m). Each plot illustrates mtDNA, with SNVs color-coded as likely benign known polymorphisms (green) or potentially suspicious variants (red). The *x*-axis represents nucleotide positions along the linear mitochondrial genome (chrM), and the *y*-axis indicates allele frequency, reflecting heteroplasmy levels. (B) Integrative Genomics Viewer (IGV) snapshot representing the alignment of sequencing reads from HG002 samples to the mitochondrial genome. The top panel shows the coverage and read alignments from the intermediate modBAM file, while the bottom panel displays the corresponding data from the modBAM-m file.

One of the main aims of MitSorter is to detect and select high-confidence mtDNA reads for accurate variant calling, as false-positive mtDNA variants can arise from misaligned NuMT-derived reads to the mitochondrial reference genome. To assess the impact of read origin on variant detection, we performed variant calling using reads extracted from the intermediate modBAM and the modBAM−m, with surprising results. Using the newly developed tool Mitopore (Dobner *et al.* 2024), calibrated on long-read sequencing data, we found that the number of variants dropped about 300-fold from a mean value of 7053 ± 3150 to 24 ± 15 when called with FASTQ files derived from intermediate and modBAM−m, respectively (Table 1, Fig. 2A). Similar results were obtained using another newly developed variant calling tool, mtDNA-Server 2 (Weissensteiner *et al.* 2024), where the number of variants dropped even more from a mean value of 9540 ± 352 to 25 ± 17 in reads derived from original and unmethylated modBAM−m, respectively (Table 1).

Results were confirmed by visualizing the respective BAM files in Integrative Genomics Viewer (IGV), where notable differences in variant density were observed, with a marked reduction in variant calls in the modBAM−m file (Fig. 2B). This supports the impact of methylation-aware processing on variant detection, particularly in differentiating between mtDNA and NuMTs. Furthermore, the drastically reduced variant load in the modBAM−m highlights the potential for false-positive variant calls arising from NuMT contamination in methylation-naïve datasets. These findings underscore the importance of MitSorter in enhancing the accuracy of mitochondrial variant calling and minimizing the confounding effects of NuMTs in genomic analyses.

## 4 Conclusion

The broad applicability of the tool lies in its ability to process ONT data without requiring modifications to standard library preparation protocols. The only essential requirement is that the DNA must remain native, ensuring accurate detection of methylation levels directly from sequencing data. Moreover, large-scale diagnostic studies are progressively adopting WGS, where both nDNA and mtDNA are analysed simultaneously (Turro *et al.* 2020), However, to date a versatile tool for accurately distinguishing the origin of sequencing reads is missing.

The precise discrimination between mtDNA and NuMTs is critical for accurate data analysis, especially in studies focused on mitochondrial diseases and phylogenetics. NuMTs, if not properly accounted for, can confound the analysis of mtDNA mutations, potentially leading to misdiagnoses or erroneous conclusions regarding mitochondrial disease mechanisms (Maude *et al.* 2019). Therefore, analysing the modBam−m offers a reliable starting point for downstream analysis, such as variant calling and mtDNA genome assembly, by ensuring a cleaner dataset devoid of confounding methylated reads. Furthermore, this tool may also be used for validating mitochondrial enrichment protocols.

Recent advancements in WGS have uncovered extremely rare NuMTs in humans, indicating that mtDNA-to-nuclear transfer is an ongoing event especially in cancers (Wei *et al.* 2022). However, the rate of NuMT formation in the germline is still not well understood. The analysis of the modBAM+m may help identify recent mtDNA insertions into nDNA (Supplementary Fig. S2, available as supplementary data at *Bioinformatics Advances* online) shedding light on the highly heterogeneous and dynamic human NuMT landscape and potentially giving new insights on diseased conditions when SVs in protein-coding genes may affect their functionality.

The tool presented here offers a valid solution for the long-standing challenge of differentiating mtDNA from NuMTs. By exploiting ONT’s unique ability to detect DNA methylation, we provide an additional layer of analysis that enhances specificity and accuracy in variant calling. This approach addresses key challenges in mitochondrial studies, particularly in the consideration of heteroplasmic variants, and opens new avenues for exploring the functional implications of NuMT methylation in nuclear genomic contexts.

Future developments may include adapting the tool to other sequencing platforms. Furthermore, expanding the dataset to diverse populations could yield broader insights into NuMT biology and its evolutionary significance.

## Supplementary Material

vbaf135_Supplementary_Data

## Data Availability

Source code and documentation are available at https://github.com/asvarvara/MitSorter.
