# Supplementary material for: MitSorter: a standalone tool for accurate discrimination of mtDNA and NuMT ONT reads based on differential methylation
Source: Bioinform Adv. 2025 Jul 10;5(1):vbaf135. doi: 10.1093/bioadv/vbaf135 (PMC12275464; doi:10.1093/bioadv/vbaf135)
Supplement: vbaf135_Supplementary_Data [file vbaf135_supplementary_data.docx]

**Online Supplementary Materials for:**

**MitSorter: a standalone tool for accurate discrimination of mtDNA and NUMT ONT reads based on differential methylation**

Sharon Natasha Cox^1*^, Angelo Sante Varvara^1*^, Graziano Pesole^1,2,3^

^1^Department of Biosciences, Biotechnology and Environment, University of Bari “Aldo Moro”. Bari, Italy

^2^ Institute of Biomembranes, Bioenergetics and Molecular Biotechnologies, National Research Council (CNR), 70126 Bari, Italy

^3^ Consorzio Interuniversitario Biotecnologie, Trieste, 34148 Italy

*These two authors contributed equally

**
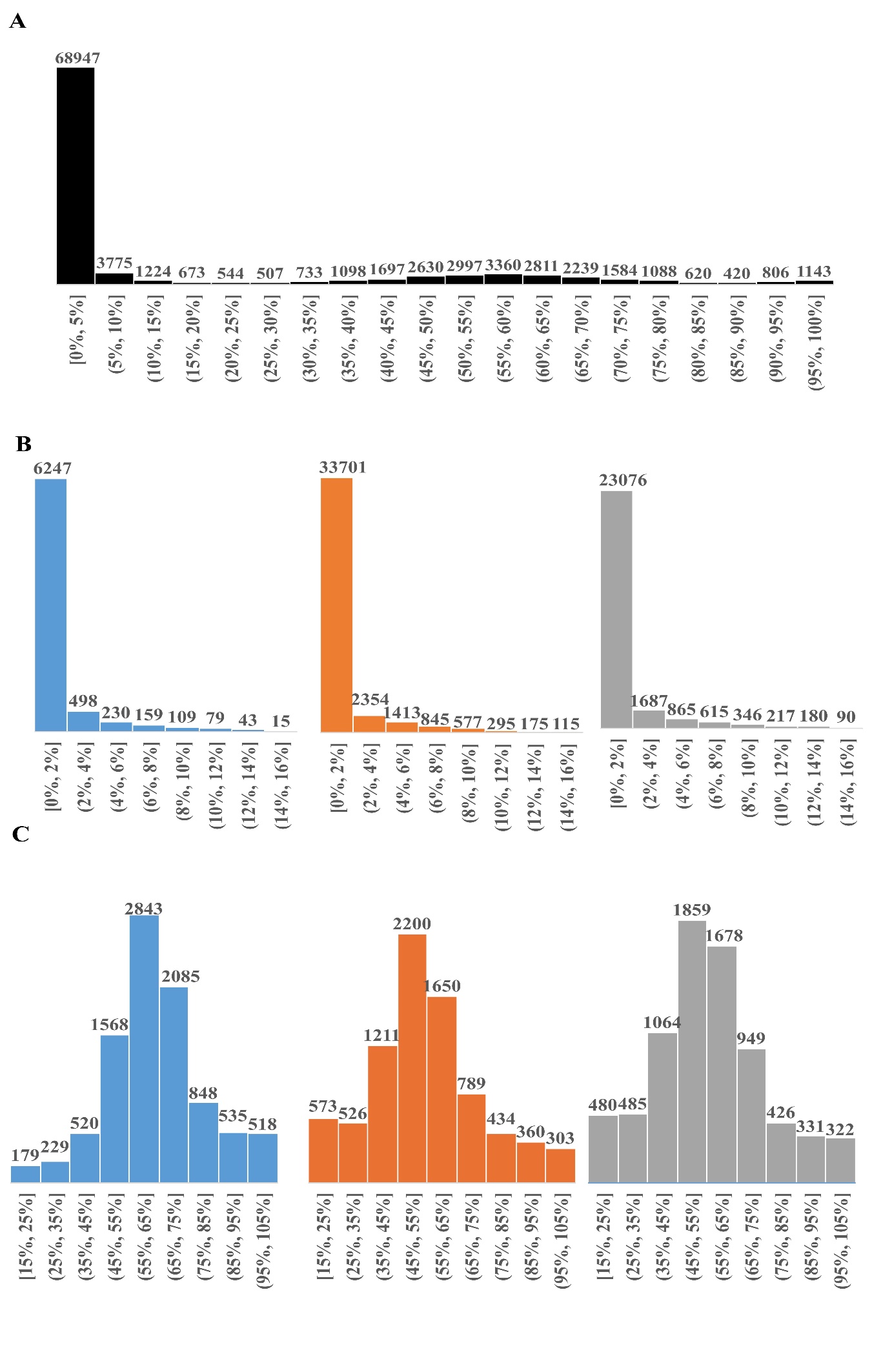
**

**Supplementary Figure 1.** **CpG Methylation Analysis of Reads Mapping to ChrM**. **A)**  A bimodal distribution of the percentage of CpG methylation is observed considering the intermediate modBAM files cumulatively. Most reads fall in the 0%-5% methylation range, likely corresponding to true mtDNA-derived reads, while the secondary peak at higher methylation levels around 55% likely corresponds to NUMTs. **B)** modBAM-m shows that the large majority of reads fall within the 2% methylation range, indicating that the filtered modBAM-m file exhibits minimal CpG methylation for the three different samples tested. **C)** modBAM+m shows a methylation level ranging from 15% to 100% with a clear peak around the 55% bin. X-axis represents methylation percentage bins, while the y-axis indicates the number of reads per bin. HG002 (blue), HG003 (orange), and HG004 (gray).


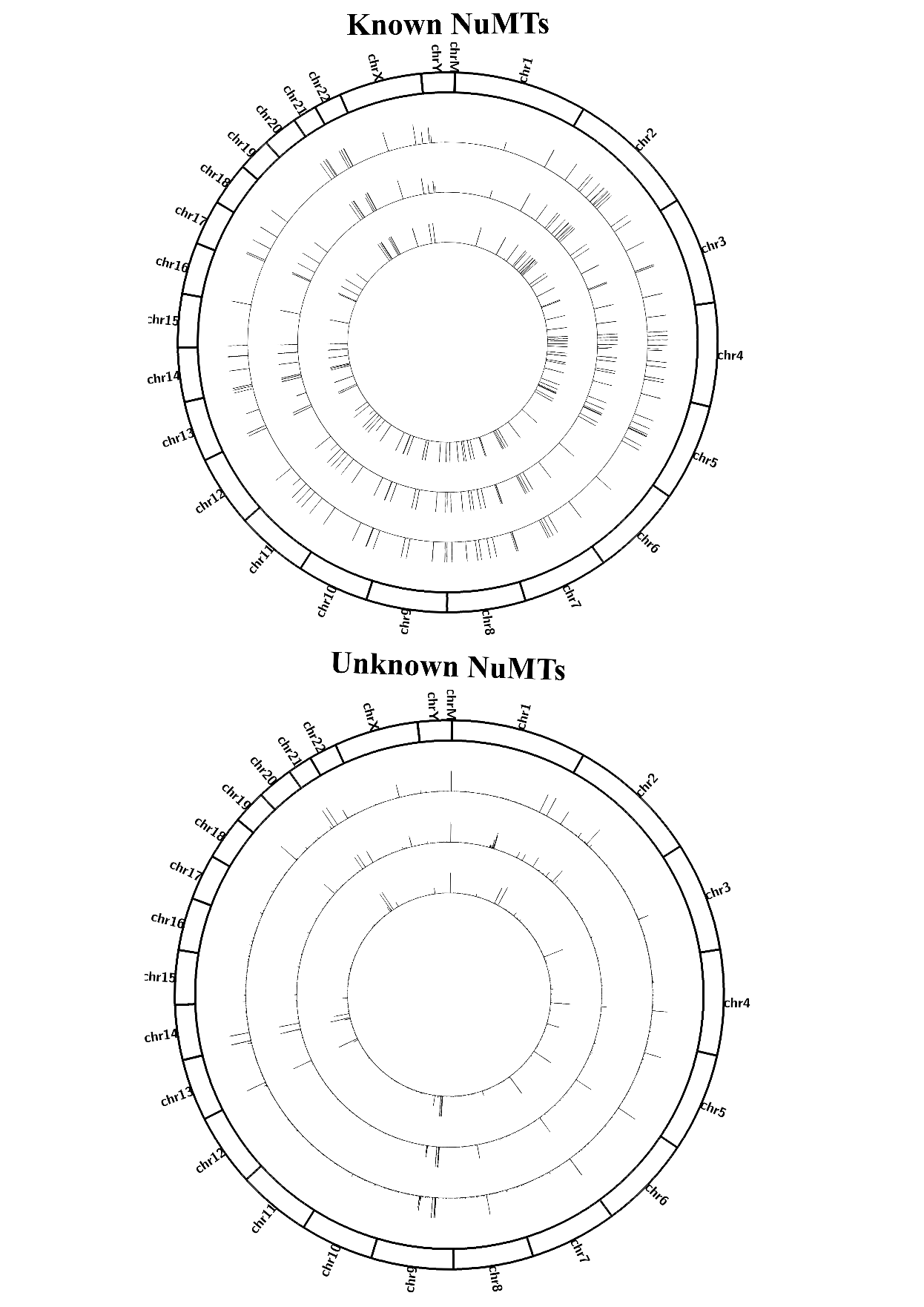


**Supplementary Figure 2.** **Coverage of modBAM+m reads across the T2T genome, highlighting known and unknown NuMT-associated reads**. To identify recent mitochondrial DNA (mtDNA) insertions into the nuclear genome (nDNA), sequencing reads were first extracted from modBAM+m files using samtools fastq. The resulting FASTQ files were aligned to the T2T-CHM13 reference genome, and the alignments were filtered using samtools view -L to retain only reads overlapping known nuclear mitochondrial DNA segments (NuMTs), as defined by Tao et al. (2023). This procedure yielded two BAM files: one containing reads mapping to known NuMT (upper plot), and another containing reads that did not overlap these regions, representing potential undocumented NuMTs (lower plot). The alignments were visualized using Circos (v0.69-8). In both circular plots, from the outermost to the innermost rings, samples HG002, HG003, and HG004 are shown. Clusters of known NuMTs are clearly observed in specific genomic regions (upper), while the lower plot highlights the distribution of reads potentially supporting novel NuMT insertions.
